# Disulfide and Fully Reduced HMGB1 Induce Different Macrophage Polarization and Migration Patterns

**DOI:** 10.3390/biom11060800

**Published:** 2021-05-28

**Authors:** Henna Salo, Heshuang Qu, Dimitra Mitsiou, Hannah Aucott, Jinming Han, Xingmei Zhang, Cecilia Aulin, Helena Erlandsson Harris

**Affiliations:** 1 Department of Medicine, Solna, Rheumatology Unit, Centre for Molecular Medicine, Karolinska Institutet, 17176 Stockholm, Sweden; henna.salo@ki.se (H.S.); heshuang.qu@ki.se (H.Q.); dimitramitsiou@gmail.com (D.M.); h.aucott@outlook.com (H.A.); cecilia.aulin@ki.se (C.A.); 2 Department of Clinical Neuroscience, Centre for Molecular Medicine, Karolinska Institutet, 17176 Stockholm, Sweden; hanjinming1202@126.com (J.H.); xingmei.zhang@ki.se (X.Z.)

**Keywords:** HMGB1, dsHMGB1, frHMGB1, macrophages, polarization, TLR4, RAGE, migration, inflammation

## Abstract

Macrophage plasticity enables cells to obtain different functions over a broad proinflammatory and repairing spectrum. In different conditions, macrophages can be induced by high-mobility group box 1 (HMGB1), a nuclear DNA-binding protein that activates innate immunity, to polarize towards a pro- (M1) or anti-inflammatory (M2) phenotype. In this study, we investigated the phenotypes of murine bone-marrow-derived macrophages (BMDMs) induced by different HMGB1 redox isoforms in depth. Our results demonstrate that disulfide HMGB1 (dsHMGB1) induces a unique macrophage phenotype that secretes pro-inflammatory cytokines, rather than inducing metabolic changes leading to nitric oxide production. Fully reduced HMGB1 (frHMGB1) did not induce macrophage polarization. The migrating function of BMDMs was measured by scratch assay after the stimulation with dsHMGB1 and frHMGB1. Both dsHMGB1 and frHMGB1 induced cell migration. We found that dsHMGB1 mediates cytokine secretion and cellular motility, mainly through toll-like receptor 4 (TLR4). Importantly, our data shows that dsHMGB1 and frHMGB1 induce distinct BMDM polarization phenotypes, and that dsHMGB1 induces a unique phenotype differing from the classical proinflammatory macrophage phenotype.

## 1. Introduction

HMGB1 is a prototypic damage-associated molecular pattern (DAMP), with its homeostatic function being DNA binding. However, HMGB1 can be released from cells in response to cellular stress or lytic cell death. Extracellular HMGB1 induces pro-inflammatory responses and cell migration [1,2,3,4]. The extracellular functions of HMGB1 have attracted a lot of attention, as HMGB1 is a major cause of excessive damage in inflammatory conditions, and has been established as a potential therapeutic target [5].

Extracellular HMGB1 initiates and regulates inflammatory responses by switching its redox status. HMGB1 contains three cysteines, which can be reduced or oxidized. As the redox status completely changes the function of HMGB1, it is crucial to take into consideration its redox isoform. If all cysteines are oxidized (HMGB1C23soC45soC106so), HMGB1 has no known proinflammatory activity [6]. When HMGB1 is fully reduced (HMGB1C23hC45hC106h, frHMGB1), it functions as a chemokine, forming a complex with the chemokine (C-X-C motif) ligand 12 (CXCL12) and interacting with the C-X-C chemokine receptor type 4 (CXCR4). The oxidation of the C23 and C45 residues leads to the formation of an intramolecular disulfide bond (HMGB1C23-C45C106h, dsHMGB1), and HMGB1 loses its ability to interact with CXCR4, instead being able to bind to TLR4 and induce cytokine production. The receptor for advanced glycation end products (RAGE) binds to all HMGB1 redox isoforms, and HMGB1–RAGE binding leads to proinflammatory responses in myeloid cells [7,8]. In healthy physiological conditions, only frHMGB1 is present in tissues not exposed to constant skeletal muscle, whereas, in inflammatory conditions, infiltrating leukocytes are the source of dsHMGB1. Tissues containing high numbers of resident leukocytes, such as spleen and liver tissues, contain dsHMGB1 even in healthy physiological conditions [3].

Macrophages are versatile myeloid cells that play an important role in tissue homeostasis, immune defense, and inflammatory progression and resolution. Macrophages enter the site of inflammation or injury either as monocytes migrating from blood or as tissue-resident macrophages inside the tissue. Activated macrophages obtain different phenotypes, which can be detected as a shift in morphology, gene expression, cellular metabolism, or functionality [9,10,11]. It is evident that such macrophage polarization plays a role in defining the outcomes of different diseases [11,12,13]. Macrophages express all three of the major HMGB1 receptors (CXCR4, TLR4, and RAGE).

Macrophages stimulated with lipopolysaccharide (LPS), a TLR4 ligand, and interferon gamma (IFN-γ) obtain a proinflammatory phenotype (M1), which is characterized by the production of reactive nitrogen and the expression of proinflammatory cytokines, including tumor necrosis factor alpha (TNF-α) and interleukin 6 (IL-6). A combination of IL-4, IL-10, and transforming growth factor beta (TGF-β) induces anti-inflammatory macrophages (M2) with wound healing properties characterized by Arg1 expression [14]. The M1 and M2 phenotypes are the endpoints of macrophage polarization in vitro, whereas in vivo macrophages obtain different polarization states in a spectrum [15], suggesting that mediators other than the classic stimulators—LPS/IFN-γ and IL-4/IL-10/TGF-β—play a role in macrophage polarization.

Current understanding of HMGB1-mediated macrophage polarization is incomplete. HMGB1 alone has been shown to induce both M1-like [16,17,18] and M2-like [19] phenotypes. Additionally, Son et al. reported that HMGB1-C1q complexes induced an M2-like phenotype [18]. Previous reports have clearly established that HMGB1 can induce macrophage polarization in vivo and in vitro, both alone and in heterocomplex with different binding partners. However, the redox status of HMGB1 is rarely defined, and there is a lack of knowledge about HMGB1 redox’s dependence on macrophage polarization. Additionally, previous studies used diverse cell culture systems, making detailed comparisons of redox-specific, HMGB1-induced polarization and receptor usage difficult. To migrate, macrophages express different receptors depending on the polarization state [20,21]. Macrophages migrate towards the frHMGB1/CXCL12 complex, but the polarization state of migratory macrophages has not been defined.

In this study, we compared the effects of the functionally active HMGB1 redox isoforms—dsHMGB1 and frHMGB1—on macrophage polarization and migration in a controlled setup. Murine bone-marrow-derived macrophages (BMDMs) were cultured and matured with macrophage colony-stimulating factor (M-CSF). Parallel stimulations were then performed with both dsHMGB1 and frHMGB1 in their endogenous states. Our findings demonstrate the importance of HMGB1 redox isoforms and receptor binding on macrophage polarization. We show that dsHMGB1 binding to TLR4 induces BMDM polarization towards a proinflammatory macrophage phenotype that differs from the classical M1 phenotype regarding migratory abilities and nitric oxide secretion. In addition, frHMGB1 did not induce BMDM polarization; however, it did induce a migratory phenotype. Our results demonstrate that BMDM polarization induced by HMGB1 is tightly regulated by HMGB1’s redox status. Moreover, BMDMs induced by dsHMGB1 and frHMGB1 are qualitatively different from the classical M1 and M2 cells.

## 2. Materials and Methods

### 2.1. Animals

C57BL/6NTac female mice were purchased from Taconic Biosciences (Leverkusen, Germany). RAGE KO mice were originally obtained from Dr. Bernd Arnold, German Cancer Research Center (Heidelberg, Germany), and were bred in-house. Animals were housed in specific pathogen-free facilities at Karolinska University Hospital with free access to water and a standard rodent chow. For isolation of bone marrow cells, the mice were euthanized between 7 and 12 weeks of age using CO_2_. All animal experimental procedures were approved by the Stockholm North Ethical Committee (dnr 18320-2017).

### 2.2. Harvesting and Culturing of BMDMs

Bone marrow cells were obtained from femurs as previously described [22]. The cells were matured into macrophages in DMEM supplemented with 10% fetal bovine serum (FBS; F7524, Sigma, St. Louis, MO, USA), 2 mM L-glutamine (Sigma), 1 mM sodium pyruvate Sigma, 22 μM β-mercaptoethanol (Gibco, Paisley, United-Kingdom), 10,000 I.U./mL Penicillin–Streptomycin (PenStrep; Sigma), and 10 ng/mL macrophage colony-stimulating factor (M-CSF; R&D Systems, Minneapolis, MN, USA) for 10 days at 37 °C with 5% CO_2_.

### 2.3. Recombinant HMGB1 Production

Recombinant HMGB1 with a calmodulin-binding protein tag was produced as previously described [23]. In order to obtain frHMGB1, all buffers were supplemented with 5 mM DTT (Biochemica, Baden-Dättwil, Switzerland). dsHMGB1 (a kind gift from Prof. Kevin Tracey’s laboratory, Feinstein Institute, Mannhasset, NY, USA) was obtained using buffers without DTT. Endotoxin levels were determined by Limulus assay, and were lower than < 2.5 EU/mg. Absence of DNA was verified using SDS-PAGE gel electrophoresis and gel red staining. Purified HMGB1 was stored in PBS ± 0.5 mM DTT.

### 2.4. Cell Culture Experiments

BMDMs were detached from the flask using Trypsin (Sigma). Cells were washed and re-seeded in 24-well culturing plates (Sarstedt, Nümbrecht, Germany) at a density of 2.5 × 10^5^ cells/well for cytokine and nitrite measurements, or at 0.5 million cells/well for gene expression analysis, in DMEM containing 1% FBS and 10,000 I.U./mL PenStrep, and rested overnight. In order to generate cells with an M1 phenotype, 100 ng/mL LPS-EK (InvivoGen, Toulouse, France) and 20 ng/mL IFN-γ (R&D Systems) were added. In order to generate cells with an M2 phenotype, 20 ng/mL each of, IL-4, IL-10, and TGF-β, were added (R&D Systems). frHMGB1 or dsHMGB1 were added at the indicated concentrations (1–9 μg/mL). PBS was included in all experiments as a negative control.

### 2.5. Cytokine and Nitrite Measurements

Cell supernatants were collected at 4, 7, and 24 h after stimulation, and cytokine concentrations were measured using murine IL-6, TNF-α, and IL-10 DuoSet ELISAs (R&D Systems) according to the manufacturer’s instructions. In order to obtain an estimation of nitric oxide (NO) secretion, the nitrite content of the cell supernatants was used as an indirect measurement and measured using modified Griess reagent (Sigma).

### 2.6. Gene Expression Analysis

RNA was isolated using the RNeasy Plus Micro Kit (Qiagen, Hilden, Germany) and reverse transcripted to cDNA using the iScript cDNA synthesis kit (Bio-Rad) according to the manufacturers’ instructions. qPCR was performed using iQ™ SYBR^®^ Green Master Mix (Bio-Rad) and run on a CFX384 Thermal Cycler according to the manufacturer’s instructions. The following KiCqStart^®^ SYBR Green^®^ mouse primers (Merck, Kenilworth, UK) were used for measuring the expression of mRNA: *B2m* (M1_B2m), *Il6* (M1_Il6), *Tnf* (M1_Tnf), *Il10* (M1_Il10), *Nos2* (M3_Nos2), *Arg1* (M1_Arg1), *Ager* (M1_Ager), *Cxcr4* (M1_Cxcr4), and *Tlr4* (M1_Tlr4). To calculate ΔΔCt values, the Ct data were normalized against the *B2m* reference gene and PBS-treated controls. Data are presented as –ΔΔCt (log fold change), i.e., negative values correspond to downregulated gene expression and positive values correspond to upregulated gene expression in the graphs. Relative expression of unstimulated cells was determined by normalizing the Ct values acquired from genes of interest against the reference gene *b2m* value using the formula: 2^ [Ct *B2m*–Ct gene of interest].

### 2.7. Cell Migration Assay

In order to assess cell mobility, scratch assays were performed. BMDMs were seeded at 5.5 × 10^4^ cells/well in IncuCyte^®^ ImageLock 96-well plates in DMEM supplemented with 10% FBS (Sigma), 2 mM L-glutamine (Sigma), 1 mM sodium pyruvate Sigma, 22 μM β-mercaptoethanol (Gibco, Paisley, United Kingdom), and 10,000 I.U./mL PenStrep (Sigma), and rested overnight. The following day, the wells were scratched using the IncuCyte^®^ WoundMaker, and the cells were immediately incubated with PBS, LPS-EK/IFN-γ, and IL-4/IL-10/TGF-β, frHMGB1, or dsHMGB1 at the indicated concentrations of 0.03–5 μg/mL (1.2–200 nM) in DMEM, supplemented with 1% FBS and L-glutamine and sodium pyruvate as described above. When using the TLR4 inhibitor CLI095 (Sigma), cells were pre-incubated with 5 μM CLI095 or with complete media containing 1% FBS for 1 h before the scratches were made. The plates were placed into the Incucyte^®^ live cell analysis system and imaged every 2 h for 48 h. At each time point, the images and relative wound density were analyzed using the Incucyte^®^ ZOOM software (2018A, Cell Migration Analysis Software Module, Essen Bioscience, Ann Arbor, MI, USA).

### 2.8. Statistical Analysis

Graphs were created using GraphPad Prism version 8.4.3 for Windows (GraphPad Software, San Diego, CA, USA). To compare the effects of treatment to the baseline expression/secretion, a one-sample *t*-test was used. To compare several treatment groups, a one- or two-way ANOVA was used, depending on the number of categorical independent variables in each experiment. The Brown–Forsythe and Welch ANOVA with Dunnett’s T3 multiple comparisons test were used when the standard deviations (SDs) in the populations were different.

## 3. Results

### 3.1. Murine BMDMs Polarize towards M1 and M2 in Response to LPS/IFN-γ and IL-10/IL-4/TGF-β, Respectively

**Figure 1:**
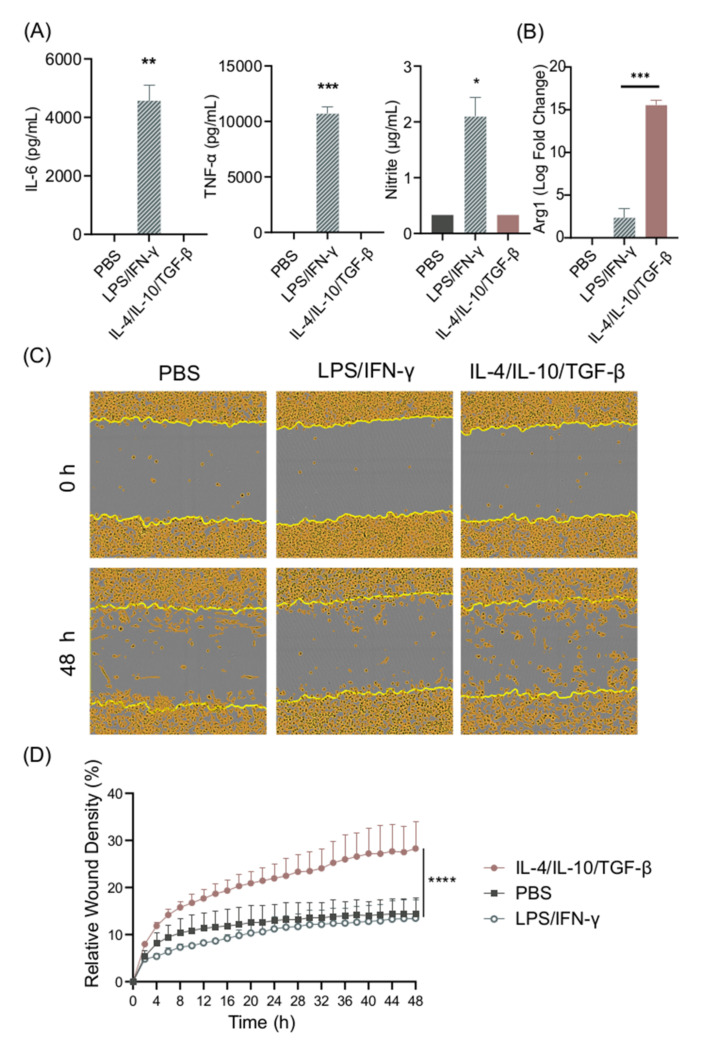
LPS/IFN-γ induce M1 polarization, while IL-4/IL-10/TGF-β induce M2 polarization. (**A**) IL-6, TNF-α, and nitrite secretion from BMDM supernatants after 24 h stimulation with LPS/IFN-γ or IL-4/IL-10/TGF-β, and (**B**) Arg1 gene expression in murine BMDMs stimulated with LPS/IFN-γ or IL-4/IL-10/TGF-β. (**C**,**D**) The cell migration ability of BMDMs stimulated with LPS/IFN-γ and IL-4/IL-10/TGF-β was measured using a scratch assay. (**C**) Representative images of scratch assay, and (**D**) the relative wound density of BMDMs stimulated with LPS/IFN-γ and IL-4/IL-10/TGF-β. Data represent n = 3 (mean + SD). Statistical analysis was performed using (**A**) a one-sample *t*-test, (**B**) *t*-test, and (**D**) a one-way ANOVA. * *p* ≤ 0.05, ** *p* ≤ 0.01, *** *p* ≤ 0.001, **** *p* < 0.0001.

In order to establish the in vitro differentiation system for BMDMs, cells were stimulated with LPS/IFN-γ or with IL-10/IL-4/TGF-β—the prototypic M1 and M2 stimuli—and their secretion of IL-6, TNF-α, and nitrite was recorded. As expected, high levels of IL-6, TNF-α, and nitrite could be detected in cell supernatants from LPS/IFN-γ-stimulated cells, whereas PBS- and IL-4/IL-10/TGF-β-stimulated cells did not secrete these pro-inflammatory cytokines (Figure 1A). IL-10 secretion, often used as a marker for M2 cells, was detected at low, non-significant levels in supernatants from LPS/IFN-γ-stimulated cells. We also measured IL-10 in cell cultures stimulated with IL-4/IL-10/TGF-β; however, the levels were below the concentration used for cell stimulation (results not shown), and thus it was not possible to distinguish between the added and secreted IL-10. We confirmed the M2 phenotype in response to IL-4/IL-10/TGF-β by measuring Arg1 gene expression, which was significantly higher in comparison to LPS/IFN-γ stimulation (Figure 1B).

Migration in response to tissue injury is an important function of M2 macrophages [21]. A scratch assay was set up in order to study the migratory and proliferative properties of BMDMs after stimulation. IL-4/IL-10/TGF-β-differentiated but not LPS/IFN-γ-differentiated cells demonstrated increased migratory features (Figure 1C,D). Cell movement was determined to be due to migration, as the cells did not proliferate at this density when cultured for 48 h (results not shown).

### 3.2. dsHMGB1 Upregulates Expression of Cytokines, but Does Not Cause BMDMs to Secrete NO

**Figure 2:**
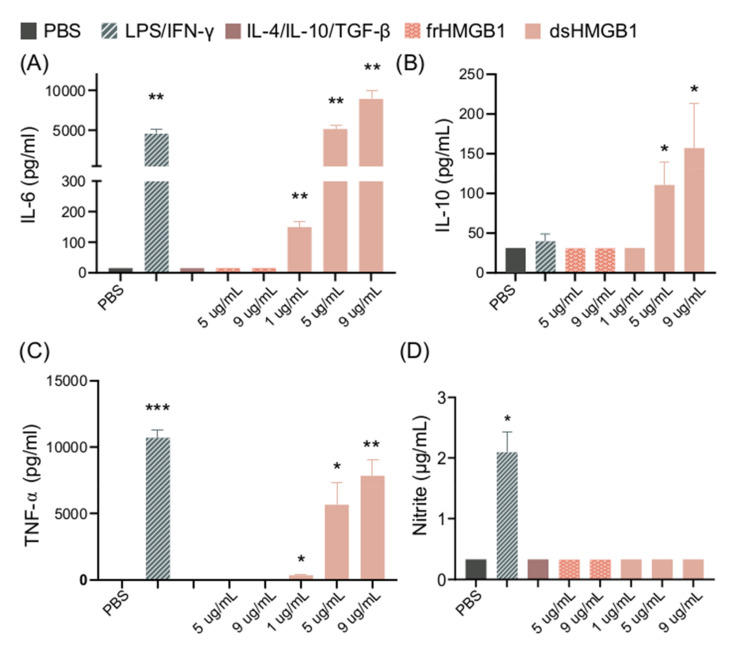
dsHMGB1 induces cytokine secretion but not nitrite production. (**A**) IL-6, (**B**) IL-10, (**C**) TNF-α, and (**D**) nitrite production by BMDMs in response to dsHMGB1 and frHMGB1 after 24 h incubation. Data represent n = 3–4 (mean + SD). Statistical analysis was performed using a one-sample *t*-test. * *p* ≤ 0.05, ** *p* ≤ 0.01, *** *p* ≤ 0.001.

BMDM cultures were stimulated with different concentrations of dsHMGB1 or frHMGB1 for 24h. dsHMGB1 stimulation induced significant release of IL-6, TNF-α, and IL-10 in a dose-dependent manner. LPS/IFN-γ stimulation induced significant release of IL-6 and TNF-α, but no IL-10 production. (Figure 2A–C). In contrast, dsHMGB1 stimulation did not induce NO production, measured as nitrite, while LPS/IFN-γ stimulation resulted in significantly increased levels of nitrite (Figure 2D). Stimulation with frHMGB1 did not result in any detectable cytokine or NO production, thus resulting in different behavior in BMDMs compared to dsHMGB1 stimulation.

### 3.3. dsHMGB1 Induces Expression of Proinflammatory Genes with a Different Kinetic Pattern Than LPS/IFN-γ

**Figure 3:**
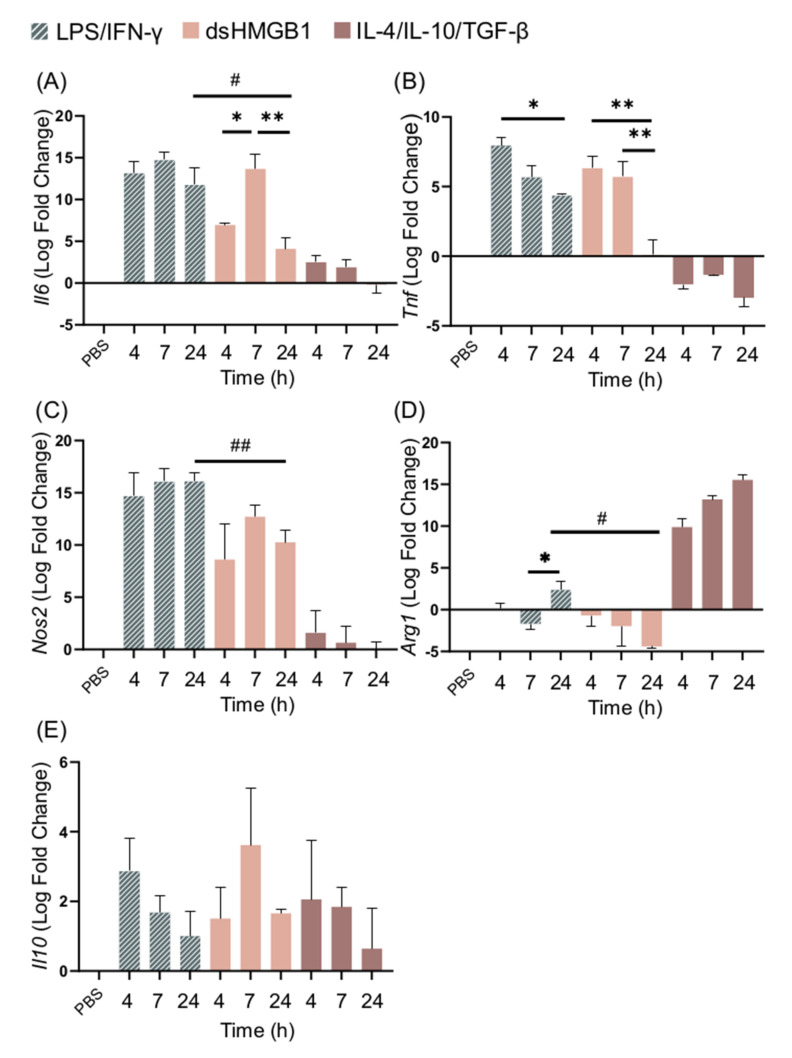
dsHMGB1 induces the upregulation of M1 signature genes, but in a different kinetic pattern to LPS/IFN-γ stimulation. Gene expression of (**A**) *Il6*, (**B**) *Tnf*, (**C**) *Nos2*, (**D**) *Arg1*, and (**E**) *Il10* in response to 5 μg/mL dsHMGB1 at different time points. Groups treated with PBS, LPS/IFN-γ, and IL-4/IL-10/TGF-β were included for comparison. Data represent n = 3. Statistical comparisons were made using a Brown–Forsythe and a Welch ANOVA with Dunnett’s T3 multiple comparisons test. * represents comparisons for the same treatment between different time points; # represents comparisons between treatments comparing the same time point. */# *p* ≤ 0.05, **/## *p* ≤ 0.01. The *p*-values of dsHMGB1 against IL-4/IL-10/TGF-β stimulation are presented in Supplementary Table S1.

Having recorded that dsHMGB1 induced a qualitative phenotype similar but not identical to that of M1 cells, we investigated the kinetics of the response. BMDM cell cultures were stimulated with LPS/IFN-γ, IL-4/IL-10/TGF-β, or dsHMGB1 for 4, 7, and 24 h, and the expression levels of *Il6*, *Tnf*, *Nos2*, *Arg1,* and *Il10* were recorded by qPCR (Figure 3A–E). dsHMGB1 stimulation resulted in *Il6* expression that peaked at 7 h and significantly decreased at 24 h when it was significantly lower than the LPS/IFN-γ-induced response. LPS/IFN-γ stimulation induced maximum expression of *Il6* after 4 h, and the expression remained high at 24 h. Both stimulations caused a similar upregulation of *Tnf* expression at 4 and 7 h. *Tnf* expression remained at 24 h with LPS/IFN-γ stimulation, while it was downregulated in cells stimulated with dsHMGB1. No kinetic differences in *Nos2* expression between LPS/IFN-γ- and dsHMGB1-stimulated cells were recorded; however, the expression was significantly stronger in LPS/IFN-γ-stimulated cells than in dsHMGB1-stimulated cells after 24 h.

*Arg1* expression was highly upregulated in IL-4/IL-10/TGF-β stimulated cells, and either downregulated or upregulated at low levels in response to dsHMGB1 and LPS/IFN-γ (Figure 3D). *Arg1* expression in dsHMGB1-stimulated cells was significantly different to IL-4/IL-10/TGF-β-stimulated cells at all of the investigated time points (Supplementary Table S1). *Il10* expression could be recorded after all three stimulations with no significant differences detected between the different stimuli. (Figure 3E).

### 3.4. frHMGB1 Does Not Polarize BMDMs

**Figure 4:**
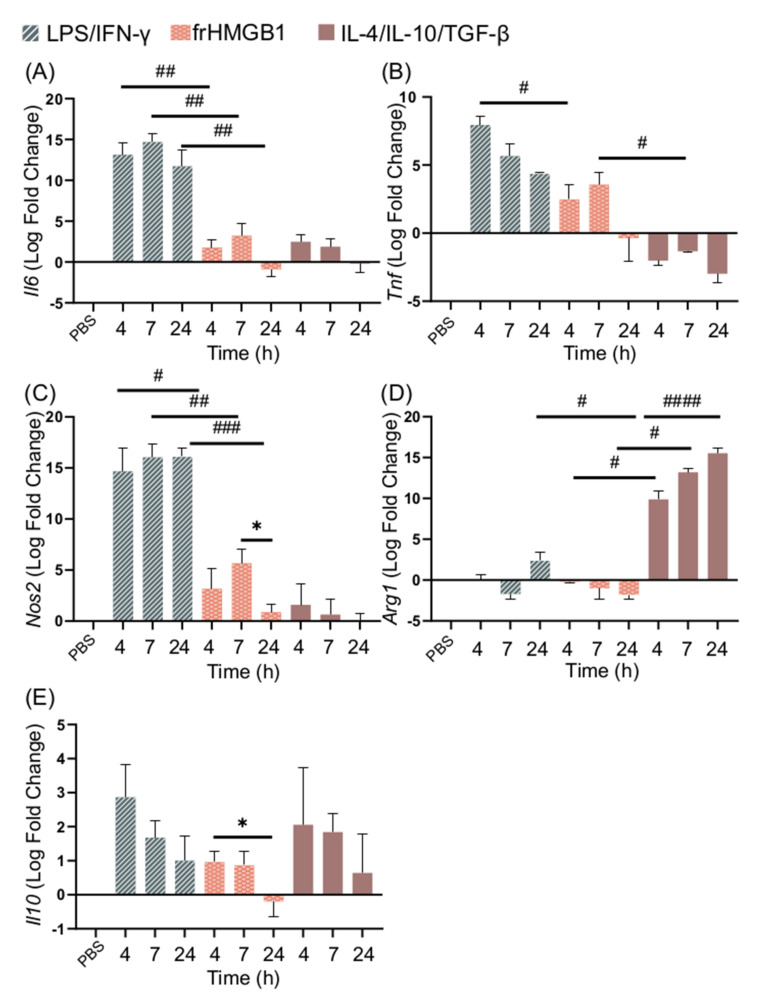
frHMGB1 induces a gene expression pattern that differs from classical M1 and M2 cells. BMDM gene expression of (**A**) *Il6*, (**B**) *Tnf*, (**C**) *Nos2*, (**D**) *Arg1*, and (**E**) *Il10* in response to 5 μg/mL frHMGB1 at different time points. Groups treated with PBS, LPS/IFN-γ, and IL-4/IL-10/TGF-β were included for comparison. Data represent n = 3. Statistical comparisons were performed using a Brown–Forsythe and a Welch ANOVA with Dunnett’s T3 multiple comparisons test. * represents comparisons for the same treatment between different time points; # represents comparisons between treatments comparing the same time point. */# *p* ≤ 0.05, ## *p* ≤ 0.01, ### *p* ≤ 0.001, #### *p* < 0.0001.

No secretion of TNF-α, IL-6, or IL-10 could be detected from frHMGB1-treated cells, indicating that frHMGB1 does not induce an M1-like phenotype (Figure 2A). To test whether frHMGB1 could induce polarization towards an M2 phenotype, the gene expression of frHMGB1-stimulated cells was determined and compared to LPS/IFN-γ and IL-4/IL-10/TGF-β stimulation. FrHMGB1 stimulation induced low upregulation of *Il6*, *Tnf*, and *Nos2*, although at significantly lower levels than LPS/IFN-γ stimulation (Figure 4A–C). *Il6* expression in response to frHMGB1 followed a nearly identical pattern to IL-4/IL-10/TGF-β stimulation (Figure 4A), although *Tnf* expression was upregulated instead of downregulated (Figure 4B). *Nos2* expression induced by frHMGB1 stimulation was significantly lower than after LPS/IFN-γ stimulation, but not significantly different from IL-4/IL-10/TGF-β stimulation. Finally, *Arg1* expression was downregulated in frHMGB1-stimulated cells (Figure 4D), in contrast to IL-4/IL-10/TGF-β-stimulated cells. *Il10* expression was not significantly different between and LPS/IFN-γ or IL-4/IL-10/TGF-β stimulation. However, *Il10* is significantly downregulated between 4 and 24 h after frHMGB1 stimulation.

### 3.5. frHMGB1 and dsHMGB1 Induce a Pro-Migratory Phenotype in BMDMs

**Figure 5:**
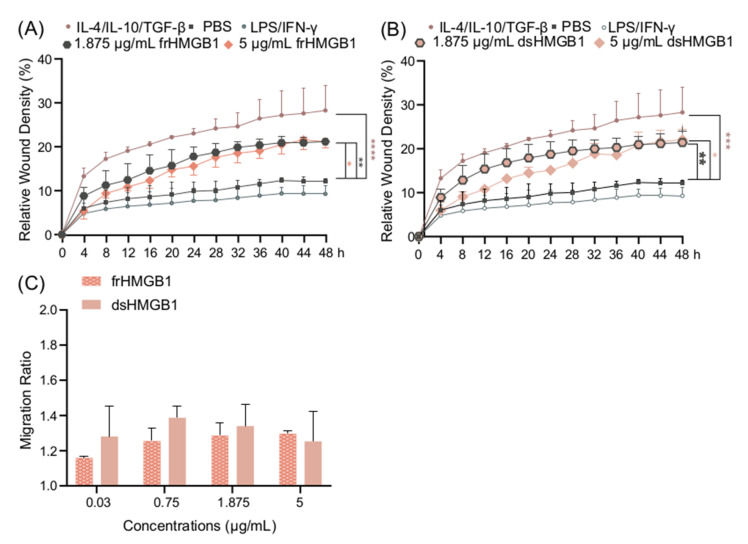
frHMGB1 and dsHMGB1 induce BMDM mobility. Confluent BMDMs were scratched and incubated with PBS, LPS/IFN-γ, IL-4/IL-10/TGF-β, frHMGB1, or dsHMGB1. The mobility changes of BMDMs after adding frHMGB1 (1.875 μg/mL or 5 μg/mL) or dsHMGB1 (1.875 μg/mL or 5 μg/mL) are shown in (**A**,**B**), respectively. (**C**) The migration ratio for BMDMs incubated with frHMGB1 or dsHMGB1 at different concentrations after 48 h. Data represent n = 3–4 (mean + SD). Statistical comparisons were performed using a two-way ANOVA on the area under the curve (**A**,**B**) or a two-way ANOVA with Sidak’s multiple comparisons. * *p* ≤ 0.05, ** *p* ≤ 0.01, *** *p* ≤ 0.001, **** *p* < 0.0001.

Since there was no indication of frHMGB1 polarizing BMDMs, and it is known that frHMGB1 can induce cell migration [6], a scratch assay was performed in order to compare the migratory effects of frHMGB1 and dsHMGB1. Both frHMGB1 and dsHMGB1 induced cell migration to a similar extent, although the effect was not as strong as the migration induced by IL-4/IL-10/TGF-β stimulation (Figure 5A,B). To investigate further, a dose titration with the different HMGB1 redox isoforms was performed. Both dsHMGB1 and frHMGB1 induced cell migration to a similar degree; no clear dose-dependent effect was observed (Figure 5C).

### 3.6. Migration and Macrophage Polarization in BMDMs Is Not Mediated via RAGE; However, dsHMGB1 and frHMGB1 Induce More Migration in the Absence of RAGE

**Figure 6:**
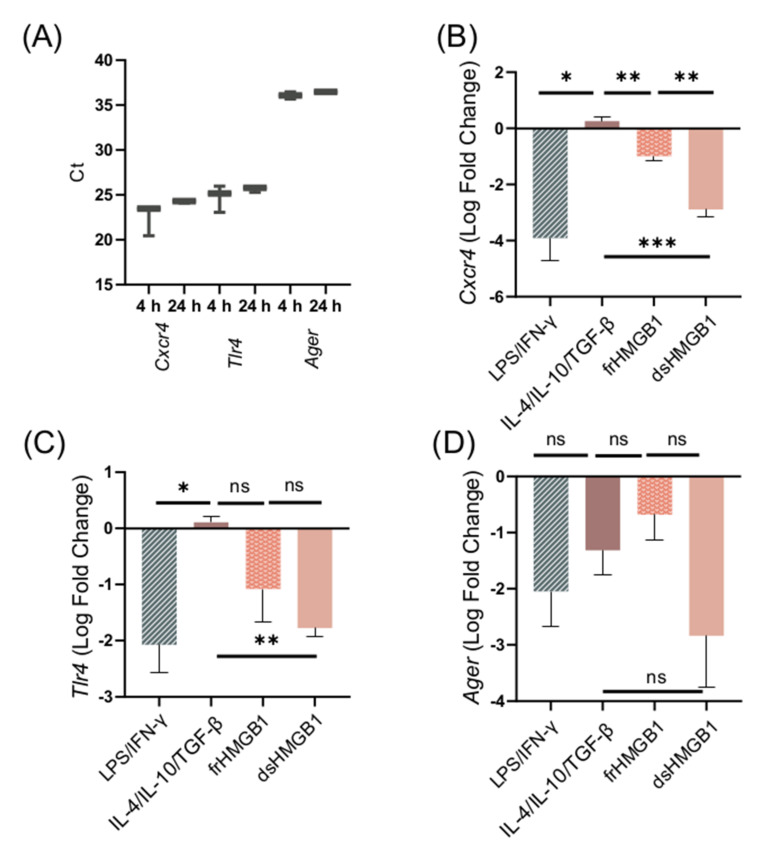
The expression of *Cxcr4*, *Tlr4*, and *Ager* in BMDMs is downregulated after dsHMGB1 and frHMGB1 stimulation. (**A**) *Cxcr4*, *Tlr4*, and *Ager* expression in unstimulated BMDMs. Data represent Ct values n = 3 (interquartile range). The gene expression of (**B**) *Cxcr4*, (**C**) *Tlr4*, and (**D**) *Ager* was determined from BMDMs incubated with LPS/IFN-γ, IL-4/IL-10/TGF-β, 5 μg/mL frHMGB1, or 5 μg/mL dsHMGB1 for 24 h. Data represent n = 3 (mean ± SD). Statistical comparisons were performed using an ANOVA and a Dunnett’s multiple comparisons test. ns: not significant; * *p* ≤ 0.05, ** *p* ≤ 0.01, *** *p* ≤ 0.001.

To further explore the mechanisms of HMGB1-induced macrophage polarization and cell migration, we investigated the dependence of the three well-characterized HMGB1 receptors CXCR4, TLR4, and RAGE. First, the expression of receptor genes in unstimulated BMDMs was explored. Expression of *Cxcr4*, *Tlr4*, and *Ager* was detected in unstimulated BMDMs at 4 and 24 h (Figure 6A). To confirm that gene expression remains the same when unstimulated cells are resting for 24 h, *Cxcr4*, *Tlr4*, and *Ager* expression was compared between 4 and 24 h (Supplementary Figure S1). There was no significant change in receptor gene expression.

Next, the effects of LPS/IFN-γ, IL-4/IL-10/TGF-β, dsHMGB1, and frHMGB1 stimulation on receptor gene expression were investigated. LPS/IFN-γ and dsHMGB1 stimulation induced a strong downregulation of all three receptors, while frHMGB1 induced a less pronounced downregulation (Figure 6B–D). IL-4/IL-10/TGF-β stimulation did not affect the expression levels of *Cxcr4* or *Tlr4*; however, it did downregulate the expression of *Ager* (Figure 6B–D). There was no significant difference in *Ager* expression between the groups (Figure 6D). In contrast, *Cxcr4* was downregulated significantly more in response to dsHMGB1 than to frHMGB1 (Figure 6B). Additionally, *Tlr4* was downregulated in LPS/IFN-γ- and dsHMGB1-stimulated cells, but was close to baseline expression in response to IL-4/IL-10/TGF-β stimulation (Figure 6C). Thus, the effect of dsHMGB1 on *Cxcr4* and *Tlr4* receptor expression was similar to the expression observed in LPS/IFN-γ-stimulated cells, and different from the expression seen after IL-4/IL-10/TGF-β stimulation. frHMGB1-induced *Cxcr4* expression was different from the expression observed in IL-4/IL-10/TGF-β- and dsHMGB1-stimulated cells.

**Figure 7:**
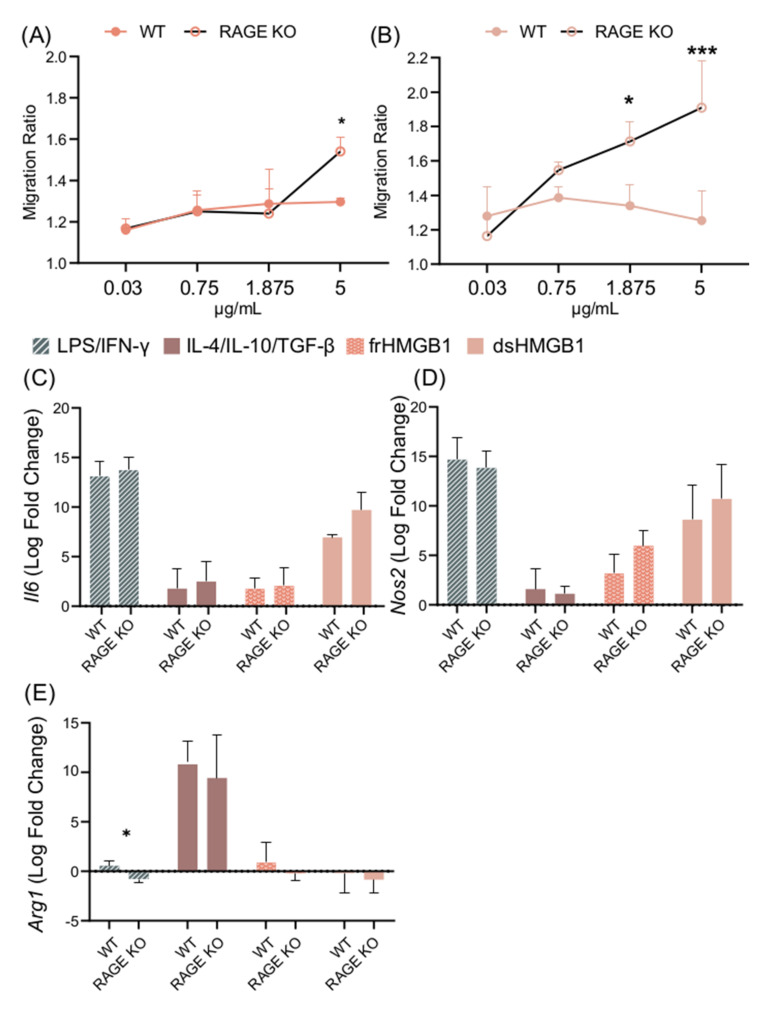
dsHMGB1 induces migration in RAGE KO BMDMs. Migration ratio for a dose titration with (**A**) frHMGB1 and (**B**) dsHMGB1 in RAGE KO and WT BMDMs. Gene expression of (**C**) *Il6*, (**D**) *Nos2*, and (**E**) *Arg1* in WT and RAGE KO BMDMs treated with PBS (negative control), LPS/IFN-γ, IL-4/IL-10/TGF-β, 5 μg/mL frHMGB1, or 5 μg/mL dsHMGB1 for 4 h. Data represent n = 3–4 (mean + SD). Statistical analysis was performed using (**A**) a two-way ANOVA with Sidak’s multiple comparisons or (**C**–**E**) a Welch’s *t*-test. * *p* ≤ 0.05, *** *p* ≤ 0.001.

After confirming the expression of *Cxcr4*, *Tlr4*, and *Ager* in BMDMs, their impact on HMGB1-induced polarization and migration was explored. RAGE was investigated first, as it is known to bind all HMGB1 redox isoforms [8]. A titration assay was performed using WT and RAGE KO BMDMs. frHMGB1 induced significantly more migration in RAGE KO BMDMs than in WT BMDMs (Figure 7A). However, the enhanced migration capacity could only be detected when 5 μg/mL frHMGB1 was used. dsHMGB1 stimulation induced a higher migration ratio in a dose-dependent manner in RAGE KO cells compared to WT BMDMs, reaching significant differences with 1.875 μg/mL and 5 μg/mL concentrations (Figure 7B). To investigate whether RAGE had an impact on HMGB1-induced macrophage polarization in BMDMs, RAGE KO BMDMs were stimulated with dsHMGB1 and frHMGB1. No differences were detected in *Il6*, *Nos2*, or *Arg1* gene expression in WT and RAGE KO BMDMs stimulated with dsHMGB1 or frHMGB1 (Figure 7C–E). M1 and M2 polarization were performed simultaneously as controls, and no differences were observed with either treatment, with the exception of the *Arg1* gene (Figure 7E), which following LPS/IFN-γ stimulation was slightly upregulated in WT cells and slightly downregulated in RAGE KO cells.

### 3.7. dsHMGB1-Induced Migratory Phenotypes Require TLR4

After observing that the dsHMGB1-induced macrophage phenotype induced higher migration in WT BMDMs than in RAGE KO cells, we hypothesized that HMGB1-induced macrophage migration might involve TLR4, and that the effect might be inhibited by RAGE.

**Figure 8:**
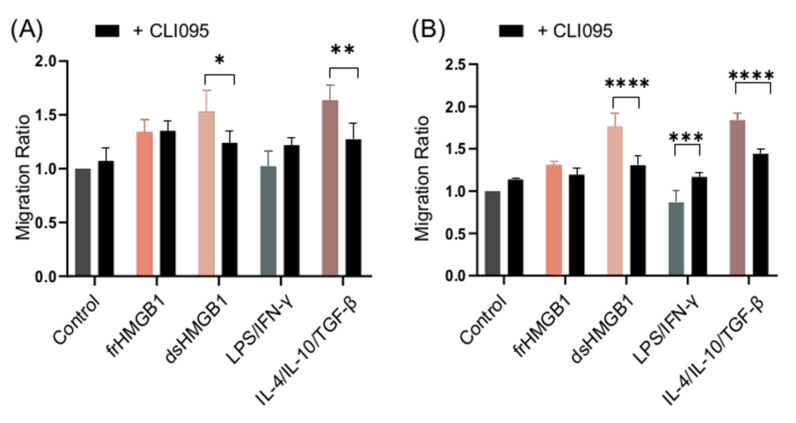
TLR4 inhibition reduces dsHMGB1-induced migration. Migration ratio data for (**A**) WT and (**B**) RAGE KO BMDMs treated with 1.875 μg/mL frHMGB1 or 1.875 μg/mL dsHMGB1 for 48 h in the presence or absence of 5 μM CLI095, a TLR4 inhibitor. Data represent n = 4 mice (mean + SD). Statistical comparisons were performed using a two-way ANOVA and Sidak’s multiple comparisons test. * *p* ≤ 0.05, ** *p* ≤ 0.01, *** *p* ≤ 0.001, **** *p* < 0.0001.

WT BMDMs were stimulated with frHMGB1 and dsHMGB1 in the presence and absence of the TLR4 inhibitor, CLI095, in order to compare and establish whether TLR4 mediates migratory functions. The functionality of the inhibitor was confirmed by measuring IL-6 and TNF-α levels in cell supernatants (Supplementary Figure S2). dsHMGB1- and IL-4/IL-10/TGF-β-stimulated cells, but not frHMGB1-stimulated cells, migrated less when TLR4 was inhibited (Figure 8A). Thus, dsHMGB1-induced migration in BMDMs involves TLR4, whereas frHMGB1 uses another receptor.

As TLR4–RAGE cooperation is important for HMGB1-induced pro-inflammatory cytokine secretion [24], an experiment was set up in order to compare the difference in migration when signaling from both receptors was inhibited. RAGE KO BMDMs were stimulated in the presence and absence of CLI095. Inhibition of TLR4 in RAGE KO BMDMs resulted in a higher reduction in the migration ratio of dsHMGB1- and IL-4/IL-10/TGF-β-stimulated cells compared to RAGE KO alone (Figure 8B), whilst LPS/IFN-γ-stimulated cells migrated more when TLR4 was inhibited in RAGE KO cells (Figure 8B).

## 4. Discussion

The inflammatory and migratory functions of extracellular HMGB1 are well established. Macrophages are important in both homeostasis and inflammation, having different functional features depending on the extracellular stimuli. As macrophage polarization in vivo is a spectrum, rather than distinct M1 or M2, there is a growing interest in characterizing the functional phenotypes mediated by immune-activating molecules such as cytokines, DAMPs, and PAMPs. The relationship between HMGB1 and macrophage polarization has been reported; however, the phenotypes induced by the different HMGB1 redox isoforms have not previously been studied in a controlled manner, nor compared to the classical M1 and M2 phenotypes. This knowledge gap prompted us to set up an in vitro system using murine BMDMs in order to compare the induction of macrophage polarization by the different HMGB1 redox isoforms to the classical M1 and M2 inducers LPS/IFN-γ and IL-4/IL-10/TGF-β, respectively.

Our study has two novel findings: We observed that dsHMGB1 induces an M1-like phenotype; however, certain features are distinct from the classical M1 phenotype induced by LPS/IFN-γ. In contrast to LPS/IFN-γ, dsHMGB1-induced BMDMs did not produce NO; however, they displayed increased cellular migration. This dsHMGB1-induced migration is mainly mediated via TLR4 and not RAGE.

When comparing the cytokine-inducing features of HMGB1, it was apparent that frHMGB1 did not induce any cytokine production, while dsHMGB1 did. Similarly to LPS/IFN-γ stimulation, dsHMGB1-induced macrophages secreted pro-inflammatory cytokines, including TNF-α and IL-6, and the anti-inflammatory cytokine IL-10. However, dsHMGB1-induced macrophages did not induce NO production. Even though dsHMGB1 induced the expression of *Nos2*, NO could not be recorded. Thus, additional stimuli may be required for NO production, and dsHMGB1 alone induced a pro-inflammatory phenotype, but one distinct from classical M1. Furthermore, dsHMGB1- and LPS/IFN-γ-stimulated BMDMs exhibited different migration ability. dsHMGB1 stimulation increased cellular motility, while LPS/IFN-γ stimulation did not. These results indicate that dsHMGB1 induces a unique M1-like macrophage phenotype.

The induction of migration by dsHMGB1 was an interesting observation, as HMGB1-induced migration has been previously demonstrated to be a feature of frHMGB1, mediated via complex formation with CXCL12 and ligation to CXCR4 [6]. The migration-inducing capacity of frHMGB1 via CXCR4 was initially demonstrated in RAGE- and TLR4-deficient BMDMs. However, the role of dsHMGB1 during this process was not previously investigated [25]. The dogma that frHMGB1 and dsHMGB1 have mutually exclusive functions was further supported by a recent study showing that primary human cardiac fibroblasts expressing CXCR4, but not RAGE or TLR4, do not migrate in response to dsHMGB1 [26]. It is important to point out that the scratch assay used for measuring migration in our study differs from the chemotaxis assay used in previous studies. Thus, the use of the scratch assay for measuring the function of migration may explain the previous overlooking of the migration-induced features induced by dsHMGB1.

RAGE was the first receptor described as binding HMGB1 in macrophages [7,27]. The HMGB1–RAGE axis has been shown to mediate cell migration, induce NF-κB activation, and promote cytokine production. Recently, an important function of RAGE in mediating the uptake of HMGB1 and HMGB1 complexes for transport into the cytosolic compartment and interaction with cytosolic sensors has been described [28]. To delineate the role of RAGE in HMGB1-induced macrophage polarization, we utilized BMDMs from RAGE-deficient mice. Cytokine production by dsHMGB1 did not differ in RAGE-deficient BMDMs when compared to WT BMDMs. The only recorded difference was the increased migratory capacity of RAGE-deficient cells when stimulated with dsHMGB1 and frHMGB1, suggesting an inhibitory role of RAGE in migratory functions. frHMGB1 mediates cell migration via CXCR4, and this is shown to be independent of RAGE [25]. Regarding dsHMGB1, the collaboration between RAGE and TLR4 is more complex. dsHMGB1 does not require RAGE when TLR4 is present on the cell surface, but RAGE has been shown to transfer TLR4 on the cell surface in BMDMs from male mice [24,25]. Differences in TLR4 signaling have been reported to differ between male and female murine macrophages [29]. This study was limited to female mice; thus, it is not clear whether the outcome we observed was sex specific.

In a previous study, HMGB1 alone was used to induce murine splenic macrophages and human monocytes to produce pro-inflammatory cytokines via RAGE and TLR4, and these were denoted to be M1 cells. However, C1q inhibited the HMGB1-induced M1 phenotype. In human monocytes, the HMGB1–C1q–RAGE complex even led to the M2 phenotype [18]. In our study, we investigated the endogenous features of HMGB1, as opposed to its features in complexes with other molecules such as C1q, which may explain why we never saw the M2 phenotype mediated by HMGB1—not to mention the possible differences between human monocytes and murine BMDMs.

Our finding that a lack of RAGE increased dsHMGB1- and frHMGB1-induced migration needs further exploration. As the concentrations of dsHMGB1 and frHMGB1 required to enhance migration effects in the absence of RAGE differed, this indicated a difference in receptor usage between the two redox isoforms. Deficiency of RAGE may alter the expression of other receptors that interact with dsHMGB1 and mediate migration. It should also be noted that the BMDMs used in our study expressed low levels of *Ager*, the gene coding for RAGE, and that the expression decreased further upon stimulation. Differential expression of RAGE in different tissue macrophages is evident; for example, alveolar macrophages have a relatively high expression of RAGE [30]. Thus, the use of low-expressing and RAGE-deficient BMDMs might preclude the detection of RAGE-mediated functions, but allowed us to perform the comparisons of HMGB1 with classical M1 and M2 inductors in a well-established in vitro culture system.

To further validate our finding that dsHMGB1 induced cellular migration, we performed stimulations with BMDMs treated with a TLR4 inhibitor. In agreement with dsHMGB1 being a TLR4 ligand and migration being induced via the NF-κB pathway [31], TLR4 blockade inhibited the migration of BMDMs. Interestingly, TLR4 blockade also significantly suppressed migration in IL-4/IL-10/TGF-β-stimulated cells. A potential explanation for this unexpected finding could be the induced release of endogenous TLR4 ligands—i.e., HMGB1—by BMDMs. Following the LPS/IFN-γ stimulation of macrophages, IL-1β and TNF-α can cause HMGB1 secretion. However, M2 cells might fail to release HMGB1 [32], and we could not detect HMGB1 in the supernatants.

In summary, dsHMGB1 and frHMGB1 induce different murine BMDM phenotypes, where dsHMGB1 induces macrophage polarization towards an M1-like phenotype; however, it differs from the classic M1 induced by LPS/IFN-γ. In addition to inducing cytokines, dsHMGB1 induces cell migration. frHMGB1 does not polarize BMDMs towards M1 or M2, although it does induce migration of BMDMs. Whereas dsHMGB1 induces both cell-migratory and cytokine-inducing functions by binding to TLR4, frHMGB1 mediated migration via other receptors. We also conclude that RAGE is not essential for the pro-inflammatory macrophage phenotype induced by dsHMGB1.

## 5. Conclusions

This is the first study in which the effects of different HMGB1 redox isoforms on macrophage polarization have been studied in parallel. Our results demonstrate that dsHMGB1 induces BMDMs towards the proinflammatory polarization spectrum, inducing TNF-α, IL-6, and IL-10 production. However, the phenotype differs from the classical M1 phenotype induced by LPS/IFN-γ, as dsHMGB1-induced BMDMs did not produce NO. Furthermore, dsHMGB1 can induce migration of BMDMs via TLR4, unlike classical M1 cells. DAMPs such as HMGB1 may exert effects on macrophage activation in a unique manner, which has significant impact on inflammatory processes, and certainly deserves further investigation.

## Data Availability

The data presented in this study are available on request from the corresponding author.

## Supplementary Materials

The following are available online at https://www.mdpi.com/article/10.3390/biom11060800/s1: Table S1: *p*-values from the Brown–Forsythe and Welch ANOVA with Dunnett’s T3 multiple comparisons tests used to compare 5 μg/mL dsHMGB1 against IL-4/IL-10/TGF-β treatment 4, 7, and 24 h after stimulation; Figure S1: Relative baseline expression of *Cxcr4*, *Tlr4*, and *Ager* normalized to B2m; Figure S2: Inhibition of TNF-α and IL-6 release in CLI095-treated BMDMs.
