# Supplementary material for: Disulfide and Fully Reduced HMGB1 Induce Different Macrophage Polarization and Migration Patterns
Source: Biomolecules. 2021 May 28;11(6):800. doi: 10.3390/biom11060800 (PMC8229957; doi:10.3390/biom11060800)
Supplement: Supplementary file 1 [file biomolecules-11-00800-s001.zip › biomolecules-1232767-supplementary.pdf]

Supplementary materials
Biomolecules

Disulphide and fully reduced HMGB1 induce different macrophage polarization and migration patterns

Supplementary Table S1. P-values of ANOVA and Dunnett's T3 multiple comparisons test

Gene	Comparison	95% confidence interval	Adjusted p-value
Il6	4 h IL-4/IL-10/TGFβ vs. 4 h dsHMGB1	-9,976 to 1,096	0,0385
	7 h IL-4/IL-10/TGFβ vs. 7 h dsHMGB1	-19,49 to -4,076	0,0074
	24 h IL-4/IL-10/TGFβ vs. 24 h dsHMGB1	-9,597 to 1,046	0,0527
Tnf	4 h IL-4/IL-10/TGFβ vs. 4 h dsHMGB1	-11,84 to -4,852	0,0020
	7 h IL-4/IL-10/TGFβ vs. 7 h dsHMGB1	-13,66 to -0,4393	0,0220
	24 h IL-4/IL-10/TGFβ vs. 24 h dsHMGB1	-7,914 to 1,753	0,0843
Il10	4 h IL-4/IL-10/TGFβ vs. 4 h dsHMGB1	-6,886 to 7,987	0,9920
	7 h IL-4/IL-10/TGFβ vs. 7 h dsHMGB1	-12,65 to 9,101	0,5523
	24 h IL-4/IL-10/TGFβ vs. 24 h dsHMGB1	-8,277 to 6,249	0,6472
Arg1	4 h IL-4/IL-10/TGFβ vs. 4 h dsHMGB1	5,546 to 15,62	0,0016
	7 h IL-4/IL-10/TGFβ vs. 7 h dsHMGB1	0,004570 to 30,35	0,0250
	24 h IL-4/IL-10/TGFβ vs. 24 h dsHMGB1	17,40 to 22,47	<0,0001
Nos2	4 h IL-4/IL-10/TGFβ vs. 4 h dsHMGB1	-22,69 to 8,666	0,1973
	7 h IL-4/IL-10/TGFβ vs. 7 h dsHMGB1	-17,84 to -6,295	0,0016
	24 h IL-4/IL-10/TGFβ vs. 24 h dsHMGB1	-15,65 to -4,793	0,0040

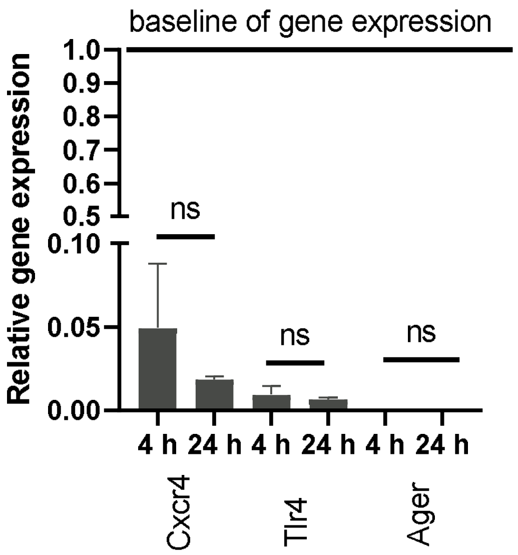

Supplementary Figure 1. Relative gene expression of Cxcr4, Tlr4 and Ager in unstimulated BMDMs. Relative gene expression was calculated by normalizing the gene of interest to expression of reference gene B2m.

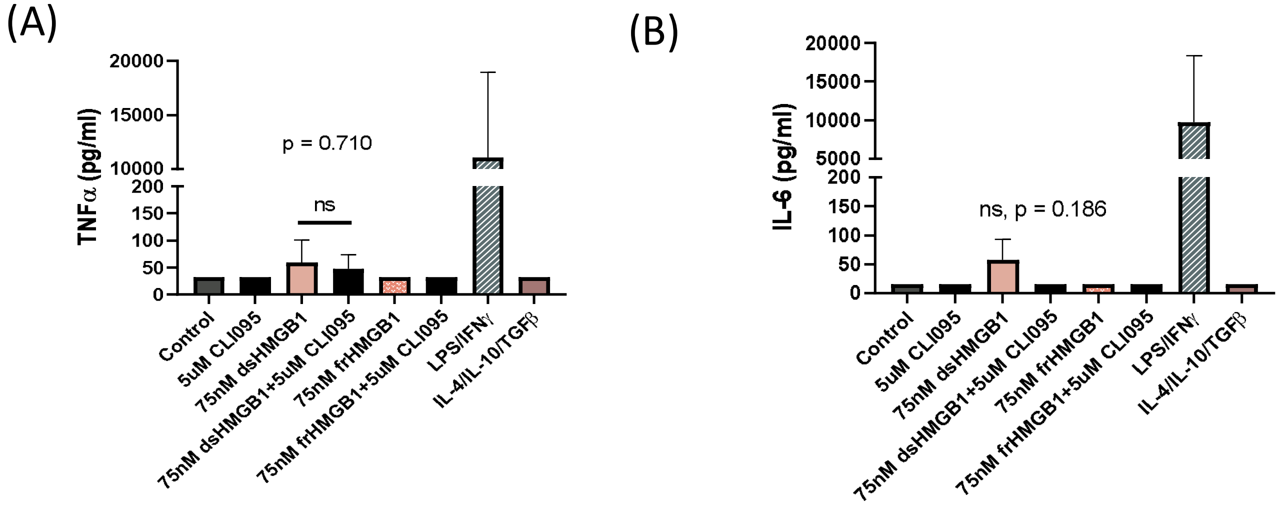

Supplementary Figure 2. Release of **(A)** TNFα and **(B)** IL-6 in response to dsHMGB1, frHMGB1, LPS/IFNγ and IL-4/IL-10/TGFβ stimulation for 24 h with and without CLI095.
